# Hyperbaric oxygen in combination with exosomes: a new strategy to promote tissue repair

**DOI:** 10.3389/fbioe.2025.1639060

**Published:** 2025-09-10

**Authors:** Shengchao Zhang, Jiaqi Zhou, Jia Liu, Tong Li, Yong Liu, Yuling Gao

**Affiliations:** Department of Rehabilitation Medicine, The First Affiliated Hospital of Dalian Medical University, College of Health-Preservation and Wellness, Dalian Medical University, Dalian, China

**Keywords:** hyperbaric oxygen, exosomes, small extracellular vesicles, tissue repair, regenerative medicine

## Abstract

Tissue repair is an essential mechanism for restoring damage caused by disease and maintaining life in organisms. Hyperbaric oxygen therapy, as a non-invasive physical treatment, has been utilized to address various tissue damage conditions. Exosomes are nanoscale vesicles released into the extracellular environment by animal cells. Their structure comprises a phospholipid bilayer membrane and includes specific functional active components, such as nucleic acids, lipids, and proteins. It can precisely modulate the behavior of target cells, restore the balance of the microenvironment, and stimulate endogenous regeneration and repair mechanisms, representing a novel tool in regenerative medicine. Evidence indicates that hyperbaric oxygen in conjunction with exosomes can markedly enhance tissue healing. Currently, there is a paucity of research about the synergistic application of the two, however they are intrinsically linked to the principles of tissue repair. Therefore, this article systematically explains the application and mechanism of hyperbaric oxygen and exosomes therapy on tissue repair, the interaction between the two, and the combined application of the two, and analyzes the safety and transformation obstacles of the combined treatment strategy, in order to provide new ideas for future combined research and clinical application.

## 1 Introduction

Nearly all ailments entail tissue injury (Liu et al., 2023). Tissue damage frequently occurs alongside vascular ischemia, inflammation, or insufficient mechanical breathing, resulting in local or systemic hypoxia (Robba et al., 2020). Hypoxia exacerbates mitochondrial structural impairment and dysfunction, activates hypoxia-inducible factor-1α (HIF-1α) along with its downstream glycolytic pathway, prompts infiltrating neutrophils and macrophages to utilize substantial quantities of oxygen and release reactive oxygen species, thereby intensifying tissue oxygen deficit. A detrimental loop of “hypoxia-inflammation” is established (Singer and Chandel, 2019; Wang et al., 2021). Concurrently, hypoxia-induced accumulation of 2-hydroxyglutarate and oxidative release of mitochondrial DNA not only impair the reparative function of regulatory T cells but also activate the NLRP3 inflammasome, hastening the production of proinflammatory mediators such as interleukin (IL)-1β and IL-18, ultimately resulting in the degradation of the extracellular matrix and the compromise of epithelial and endothelial barriers. Severe instances may result in irreparable tissue damage (Singer and Chandel, 2019). Tissue repair is a process in which the body reinstates its structure and function via a series of synergistic physiological processes following tissue injury. The significance of tissue repair is evident in sustaining bodily homeostasis, reinstating functionality, and preventing further injury (Wilkinson and Hardman, 2020). Tissue repair and regeneration is a complex and intricate process characterized by several dynamic events (Fernández-Guarino et al., 2023), including the hemostasis phase, inflammatory phase, proliferation phase, and remodeling phase (Peña and Martin, 2024). Initially, the coagulation cascade is initiated, leading to the accumulation of platelets and coagulation factors near the injury site, which release chemicals that facilitate blood clot formation. Simultaneously, immune cells secrete, invade, and polarize to activate the immune response and eliminate necrotic tissue by the release of inflammatory mediators and other processes. In the proliferative and remodeling stages, basal cells, fibroblasts, myofibroblasts, and vascular endothelial cells undergo proliferation and migration to facilitate tissue healing. Oxygen is essential for the repair of damaged tissues, particularly during the inflammatory phase (providing energy for immune cells), the proliferation phase (facilitating cell proliferation, angiogenesis, and collagen production), and the remodeling phase (assisting in collagen remodeling and death). Oxygen facilitates all facets of restoration by supplying energy and engaging in essential metabolic activities (Castilla et al., 2012; Hunt et al., 2024). Conventional tissue repair methods encompass debridement, growth factor therapy, and similar interventions. As medical technology advances, novel biological techniques have evolved, facilitating a transition from passive repair to active regeneration of tissues (McKinley et al., 2023).

Hyperbaric oxygen treatment is extensively utilized in clinical practice to enhance tissue repair owing to its oxygen-delivery capabilities; nevertheless, problems such as oxygen-induced lung injury constrain its effectiveness (Ortega et al., 2021). Exosomes therapy, an innovative non-cellular treatment in recent years, has demonstrated considerable improvements in tissue restoration (Tan et al., 2024). Recent investigations indicate that exosomes pretreatment can markedly diminish hyperbaric oxygen-induced lung injury, therefore decreasing the incidence of problems (Braun et al., 2018; Shi et al., 2025). Consequently, the integration of the two is anticipated to augment tissue healing efficacy and enhance safety, presenting significant opportunities for clinical revolution. Currently, certain research have demonstrated that the amalgamation of the two treatments exhibits significant synergistic effects in rat models of diabetic wounds, neurological disorders, and vascular diseases, and even surpasses the efficacy of each intervention alone in neurological disorders (Amiri et al., 2025; Cheshmi et al., 2023; Hjazi et al., 2024; Jafari et al., 2023; Shyu et al., 2019; 2020; Yin et al., 2024). Therefore, we hypothesize that potential links may exist between hyperbaric oxygen and exosomes. So, we examine the mechanisms of tissue repair and the potential association between hyperbaric oxygen and exosomes, alongside research in pertinent disease models, while assessing the safety and clinical translation challenges of the combined treatment strategy, with the aim of offering novel insights in the domain of tissue repair.

## 2 Hyperbaric oxygen

### 2.1 Overview of hyperbaric oxygen

Hyperbaric oxygen therapy is an innovative treatment modality that involves the breathing of pure oxygen or oxygen at elevated concentrations under atmospheric pressure greater than that of the surrounding environment. The mechanism of action primarily involves creating a high-pressure, high-oxygen environment for the damaged tissue, directly enhancing the hypoxic condition, promoting angiogenesis and collateral circulation, and improving the blood oxygen supply to ischemic tissue (Damineni et al., 2025). It can augment the bactericidal capacity of leukocytes by elevating reactive oxygen species generation (Damineni et al., 2025), and modulate the equilibrium of inflammatory mediators and oxidase activities to exert anti-inflammatory and antioxidant effects (Cannellotto et al., 2024). Moreover, hyperbaric oxygen therapy can stimulate cellular metabolism and regenerative capacity, facilitating tissue remodeling (Mackay et al., 2025). Hyperbaric oxygen therapy, recognized for its high safety, ease of operation, low cost, and non-invasive nature, has been endorsed by the International Society of Underwater and Hyperbaric Medicine for the treatment of 14 clinical conditions, including refractory wounds, thermal burns, gas gangrene, and other tissue damage disorders, establishing it as a significant adjunctive therapy for tissue repair (Ortega et al., 2021).

### 2.2 The mechanism of hyperbaric oxygen promoting tissue repair

Hyperbaric oxygen therapy facilitates tissue repair via multiple processes, primarily by enhancing cellular function and expediting the healing process through elevated partial pressure of oxygen in the tissue. Hyperbaric oxygen markedly elevates the dissolved oxygen levels in the blood by creating high-pressure and hyperoxic conditions within an enclosed environment. The elevation of dissolved oxygen under high-pressure conditions can enhance the oxygen levels in local wound tissue, including areas not reachable by red blood cells, thereby satisfying cellular oxygen requirements and accelerating tissue metabolic activity, thus facilitating wound healing (Cannellotto et al., 2024). Secondly, hyperbaric oxygen facilitates the healing and regeneration of injured tissues by stimulating angiogenesis and collagen production. Research indicates that hyperbaric oxygen facilitates angiogenesis in wound healing and enhances local oxygen levels, consequently stimulating fibroblast proliferation and collagen synthesis (Gupta and Rathored, 2024). Oxygen serves as a metabolic substrate and a signaling molecule that modulates cellular activity, participating in glycoprotein manufacturing during cell growth (Zoneff et al., 2024). During cell maturation or remodeling, oxygen participates in the synthesis of fibrous collagen as a cofactor for hydroxyproline, followed by the hydroxylation of prolyl, which acts as a cosubstrate under the catalysis of prolyl hydroxylase. Consequently, it facilitates the cross-linking of collagen molecules and improves tissue stability (Salo and Myllyharju, 2021).

Moreover, hyperbaric oxygen can diminish tissue damage by decreasing the adhesion of inflammatory cells, preventing lipid peroxidation, and exhibiting antibacterial capabilities. Research indicates that hyperbaric oxygen can enhance the expression of antioxidant enzymes (such as catalase, superoxide dismutase), anti-apoptotic proteins (such as B-cell lymphoma-2, silent information regulator related enzyme 1), and intercellular junction proteins (such as β-catenin, tight junction protein 1, occludin). Down-regulating inflammatory mediators (such as tumor necrosis factor-alpha (TNF-α) and IL-1β) and apoptosis-related proteins (such as caspase-3) to mitigate the inflammatory response, augment antioxidant capacity, and diminish cell apoptosis has demonstrated beneficial effects in expediting tissue repair (Capó et al., 2023; Ortega et al., 2021; Tejada et al., 2019). Simultaneously, hyperbaric oxygen therapy exhibits enhanced antibacterial activities, particularly against anaerobic bacteria and illnesses associated with biofilms. The body generates reactive oxygen species that are detrimental to bacteria, capable of infiltrating the bacterial cell wall, resulting in cellular damage and bacterial mortality (Cannellotto et al., 2024). The interplay of these systems renders hyperbaric oxygen therapy an excellent complement for enhancing complex wound healing and augmenting the reparative ability of injured tissues.

## 3 Exosomes

### 3.1 Overview of exosomes

Exosomes are extracellular vesicles released by cells, measuring approximately 40–200 nm in diameter, and containing diverse substances, including complex RNAs and proteins. They exhibit significant potential in intercellular communication, disease diagnosis, and treatment, emerging as a frontier in recent research on non-cellular therapies for regenerative medicine (Krylova and Feng, 2023). In addition to the remarkable regenerative and immunomodulatory capabilities of stem cells, exosomes generated from stem cells have simpler structure, higher stability, larger yield, easier storage and transportation, and lower immunogenicity, which are key carriers to replace stem cell therapy (Tan et al., 2024). Numerous research indicate that exosomes have effective reparative properties on injured tissues in dermatological conditions (including diabetic wounds, psoriasis, and certain dermatitis) and neurological disorders (such as neurodegenerative diseases or spinal cord injuries) (Wang and Yang, 2023; Yu et al., 2024). The latest report indicates that exosomes derived from mesenchymal stem cells can diminish the expression of inflammatory genes in pulmonary oxygen toxicity induced by hyperbaric oxygen, potentially aiding in the development of novel strategies to decelerate or even reverse the progression of hyperbaric oxygen injury in patients (Shi et al., 2025). Exosomes, with their exceptional properties and benefits, exhibit significant potential in tissue repair and regeneration, paving a novel pathway for the treatment of many tissue damage disorders.

### 3.2 The mechanism of exosomes promoting tissue repair

The mechanism of exosomes in tissue repair mainly includes the following aspects. Exosomes can modulate the polarization of immune cells, thereby diminishing the inflammatory response and creating a favorable milieu for the healing of injured tissues (Wang et al., 2025). Studies demonstrate that exosomes produced from adipose-derived mesenchymal stem cells (ADSCs-Exos) promote tissue repair by altering macrophage polarization from the pro-inflammatory M1 phenotype to the anti-inflammatory M2 phenotype (Wang et al., 2025). Secondly, exosomes are abundant in several growth factors that might facilitate angiogenesis and tissue regeneration in injured tissues (Hade et al., 2021). Exosomes generated from bone marrow mesenchymal stem cells (BMSC-Exos) have demonstrated the ability to regulate angiogenesis and facilitate bone tissue regeneration through the release and transfer of miR-29a. BMSC-derived exosomes enhanced tendon-bone healing by facilitating angiogenesis in rotator cuff repair (Chen et al., 2025). Third, exosomes can expedite tissue repair by transporting particular miRNAs or proteins that enhance fibroblast proliferation and collagen formation (Xiong et al., 2023). Research demonstrates that BMSC-Exosomes significantly enhanced the expression of collagen I and III in skin fibroblasts and promoted skin wound healing via the upregulation of miR-542-3p (Xiong et al., 2023). In addition, non-coding RNAs in exosomes play an equally important role in regulating cell proliferation and tissue remodeling (Gowtham and Kaundal, 2025). Fourth, exosomes can also reduce fibrosis and scarring by regulating extracellular matrix synthesis and remodeling (Baral et al., 2021). Research indicates that exosomes produced from mesenchymal stem cells (MSC-Exos) can impede skin fibrosis and diminish tissue scarring in mice through the delivery of miR-196b-5p (Baral et al., 2021). The research revealed that ultrasound-pretreated BMSC-Exosomes modulated chondrogenic and anti-adipogenic pathways, enhanced cartilage matrix regeneration, and facilitated fibrocartilage maturation via the targeted delivery of miR-140 in rotator cuff repair (Wu et al., 2024). Exosomes from human foreskin cells modulate collagen synthesis and fibrocyte differentiation in skin wound healing by stimulating the ERK/MAPK pathway, hence diminishing scar formation (Sağraç et al., 2024). Furthermore, ADSCs-Exosomes facilitated the swift restoration of corneal architecture by diminishing oxidative stress, consequently improving cell viability, decreasing cell apoptosis, and boosting the migration of corneal epithelial cells (Ma et al., 2025).

Exosomes can transport medications across the blood-brain barrier for central nervous system disorders. It has demonstrated significant potential in treating central nervous system disorders by facilitating the regeneration and repair of nerve tissue, positioning it as a promising target for such treatments (Wang and Yang, 2023). In summary, exosomes exhibit significant tissue repair capabilities through multiple pathways, including the modulation of inflammatory responses, facilitation of angiogenesis, regulation of cell proliferation and collagen synthesis, inhibition of fibrosis and scar formation, and targeted medication delivery.

## 4 Biological mechanism of hyperbaric oxygen combined with exosomes to promote tissue repair

### 4.1 Hyperbaric oxygen induced exosomes release

**FIGURE 1:**
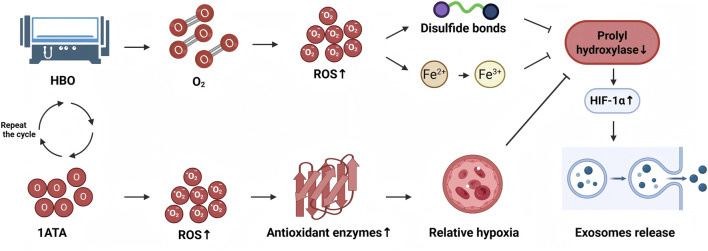
The excessive generation of reactive oxygen species from hyperbaric oxygen can oxidise critical cysteine residues in the catalytic domain of prolyl hydroxylase, leading to conformational alterations and subsequent inactivation of the enzyme. Additionally, it can oxidise Fe^2+^ in the active site of prolyl hydroxylase to Fe^3+^, resulting in the loss of the reduced cofactor necessary for catalysis and stabilisation of HIF-1α. Conversely, persistent exposure to hyperbaric oxygen consistently elevates the production of reactive oxygen species and stimulates the body to generate additional antioxidant enzymes. The half-life of antioxidant enzymes significantly exceeds that of reactive oxygen species, resulting in the “hyperoxia-hypoxia paradox” upon reversion to normoxia following hyperbaric oxygen exposure, which induces a relative hypoxic condition. The inhibition of prolyl hydroxylase activity prevents the hydroxylation and degradation of HIF-1α, resulting in its buildup.

The secretion of exosomes is a multifaceted biological process necessitating several membrane fusions (Krylova and Feng, 2023). A hypoxic environment can enhance the secretion of exosomes from cells. Muñiz-García et al. (2022) demonstrated that the exosomal release from human embryonic kidney cells was markedly elevated following hypoxia induction, correlating with the heightened expression of HIF-1α under hypoxic circumstances. Wang et al. (2014) indicated that HIF-1α enhanced Rab22a expression following hypoxic treatment of breast cancer cells, resulting in an almost twofold increase in released exosomes. A separate study by Dorayappan et al. (2018) indicated that HIF-1α facilitated the upregulation of Rab27a and the downregulation of Rab7, lysosomal membrane-associated proteins 1 and 2, and neuraminidase-1 in ovarian cancer cells subjected to hypoxia. Despite the hyperbaric oxygen environment offering elevated oxygen concentration and partial pressure, which theoretically should suppress HIF-1α activity through prolyl hydroxylase activation resulting in degradation (Fiorini and Schofield, 2024), it frequently elicits a response akin to hypoxic circumstances in practice. As shown in Figure 1, on the one hand, the excessive accumulation of reactive oxygen species produced by hyperbaric oxygen can not only oxidize key cysteine residues in the catalytic domain of prolyl hydroxylase (such as the formation of disulfide bond between Cys201 and Cys208), causing conformational change of the enzyme and inactivation (Bae et al., 2024), but also oxidize Fe^2+^ to Fe^3+^ in the active center of prolyl hydroxylase (Chandel et al., 2000). The enzyme loses the reduced cofactor required for catalysis (Chandel et al., 2000), thereby stabilizing HIF-1α. On the other hand, cellular oxygen sensing is based on fluctuations in oxygen concentration rather than absolute values. When hyperbaric oxygen therapy restores cells to normoxia by occasional hyperoxia, the cells will display a condition of relative hypoxia, hence activating regeneration pathways often induced by actual hypoxia. This occurrence is referred to as the “hyperoxia-hypoxia paradox” (Hadanny and Efrati, 2020). Repeated exposure to hyperbaric oxygen enhances the generation of reactive oxygen species and stimulates the synthesis of antioxidant enzymes, including glutathione. The half-life of antioxidant enzymes much exceeds that of reactive oxygen species, leading to a marked decrease in the ratio of reactive oxygen species to antioxidant enzymes upon reestablishment of normoxia following hyperbaric oxygen exposure. The inhibition of prolyl hydroxylase activity prevents the hydroxylation and degradation of HIF-1α, resulting in its buildup. The stability and expression level of HIF-1α facilitate the release of exosomes (Muñiz-García et al., 2022). The “hyperoxia-hypoxia paradox” exemplifies the paradoxical mechanism of exosomes release generated by hyperbaric oxygen. In a hyperoxia exposure model of neonatal lung damage, the transition to normoxia following hyperoxia treatment initiated the creation of multivesicular bodies in mast cells and stimulated exosomes release (Veerappan et al., 2016). Simultaneously, clinical evidence from Siewiera et al. (2024) shown that serum exosomes levels in patients with idiopathic acute sensorineural hearing loss dramatically increased following hyperbaric oxygen treatment (*p* < 0.05). This study’s results underscore the therapeutic importance of the “hyperoxia-hypoxia paradox” and offer a novel perspective on the mechanism of hyperbaric oxygen, suggesting that it may function via modulating exosomes levels. Consequently, exosomes are implicated in hyperbaric oxygen’s facilitation of illness recovery, and variations in exosomes levels may serve as a possible biomarker for the treatment’s success.

### 4.2 Hyperbaric oxygen affects the contents of exosomes

Prior research has demonstrated that hypoxic treatment of mesenchymal stem cells alters the mix of microRNAs and proteins in exosomes generated by these cells, hence augmenting the therapeutic potential of the exosomes (Zhuo et al., 2024). Shyu et al. (2019) observed alterations in the expression of RNA fragments and angiogenic factors in exosomes from human coronary artery endothelial cells (HCAECs) subjected to hyperbaric oxygen therapy, attributed to the relative hypoxic effects of the “hyperoxa-hypoxia paradox”. Research indicates that hyperbaric oxygen markedly enhances the expression of metastasis-associated lung adenocarcinoma transcript 1 (MALAT1) in exosomes produced from HCAECs. MALAT1, a long non-coding RNA, directly targets vascular endothelial growth factor receptor 2, regulates endothelial cell activity, promotes angiogenesis (Liu et al., 2022), and inhibits miR-92a, which is partially complementary to MALAT1 and influences angiogenesis. Consequently enhancing the expression of the angiogenic factor Kruppel-like factor 2 (KLF2) (Kattih et al., 2024). Following exposure of HCAECs to varying levels of absolute atmospheric (ATA) pressure in a hyperbaric oxygen environment, it was shown that at 2.5 ATA, the expression of MALAT1 in both HCAECs and HCAECs-derived exosomes was maximized. The peak effect was observed after 2 days of exposure and returned to baseline levels by day six. The diminished expression of MALAT1 in HCAECs and exosomes produced from HCAECs may result from elevated oxygen partial pressure or a hyperbaric oxygen environment below 2.5 ATA, which fails to sufficiently induce relative hypoxia in the cells.

Moreover, another research has demonstrated that hyperbaric oxygen can influence the expression of inflammation-associated genes in exosomes (refer to Table 1). Yin et al. (2024) utilized PCR to demonstrate that hyperbaric oxygen treatment resulted in the downregulation of several inflammation-related genes in exosomes, including *TLR5*, *FAAH2*, *SLC11A1*, *EGR3*, and *SRPK3* (all P < 0.01) and *EGR1* (P < 0.001). Whole genome sequencing was conducted on exosomes obtained from human umbilical vein endothelial cells, both without and with hyperbaric oxygen treatment. Following comparison, functions and pathways of many biological components were identified as enriched in exosomes subsequent to hyperbaric oxygen treatment (refer to Table 1). Engaged in the MHCII biosynthetic process, transport of basic amino acids, development of primary sexual characteristics, synaptic assembly, intrinsic membrane components, DNA alkylation, DNA methylation, and pathways related to the development of primary male sexual characteristics and the reproductive system, the reproductive system development pathway exhibited the highest level of enrichment. Hyperbaric oxygen regulates gene expression and signaling pathways in exosomes, significantly enhancing fibroblast proliferation and expediting the healing of diabetic wounds (Yin et al., 2024).

**TABLE 1:** The expression of related contents in exosomes was changed under hyperbaric oxygen.

Effects of hyperbaric oxygen on the contents of exosomes	
Form	Specific changes
Inflammation-related gene expression	*TLR5*↓, *FAAH2*SL↓, *C11A1*↓, *EGR3*↓, *SRPK3*↓, *EGR1*↓
Correlation function enrichment analysis	MHC class II biosynthetic processes ↑, basic amino acid transport ↑, development of primary sexual characteristics ↑, synapse assembly ↑, intrinsic membrane components ↑, DNA alkylation ↑, DNA methylation ↑, development of primary male sexual characteristics ↑, developmental pathways of the reproductive system ↑
RNA fragment	MALAT1↑, miR-92a↓
transcription factor	KLF2↑

### 4.3 Protective effects of exosomes on hyperbaric oxygen-induced injury

**FIGURE 2:**
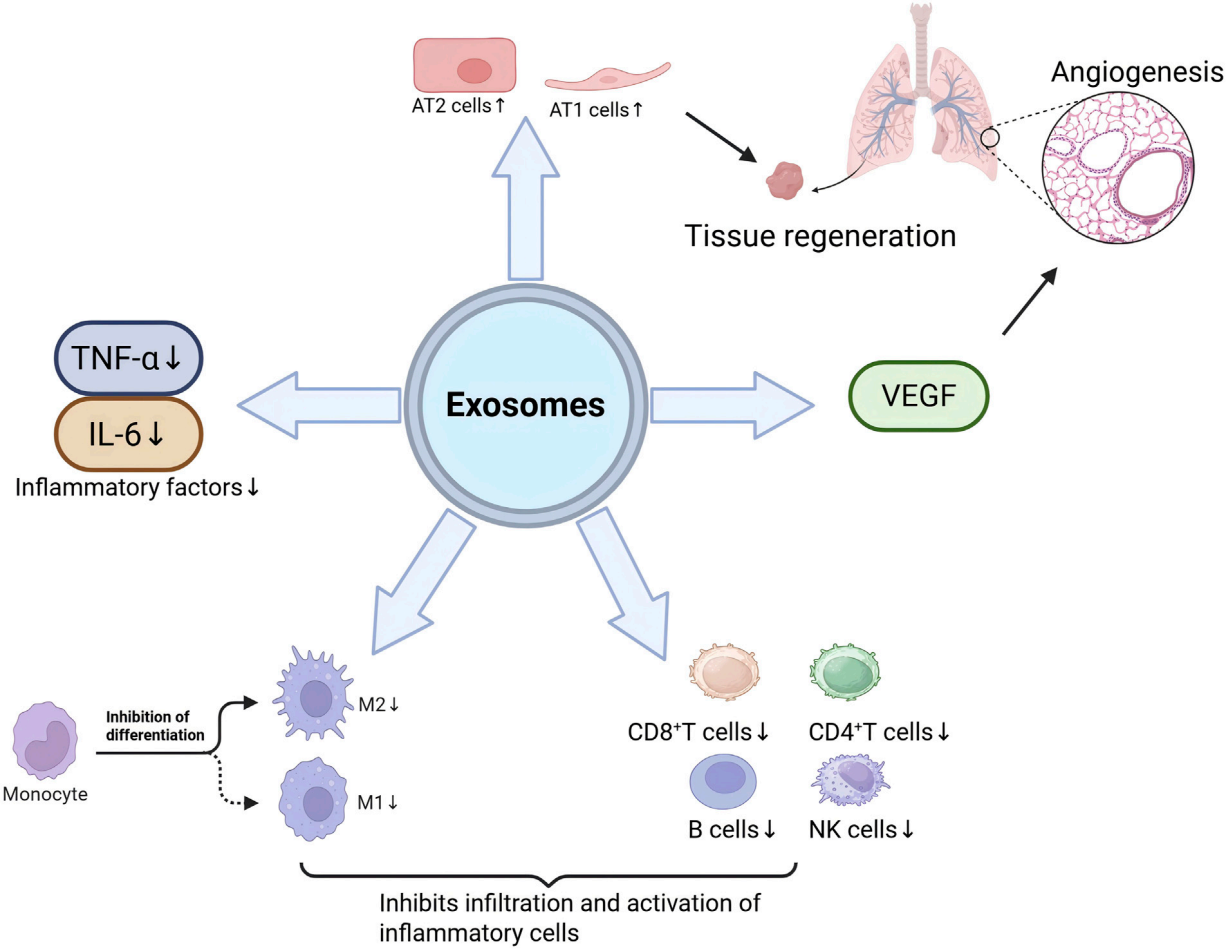
Exosomes exert a protective effect against hyperbaric oxygen-induced injury through multiple mechanisms: (1) Inhibition of macrophage M1/M2 polarization downregulates the inflammatory cascade response, thereby exerting a significant anti-inflammatory effect. (2) Exosomes drive neoangiogenesis by carrying and targeting delivery of vascular endothelial growth factor, which activates the tubular network formation potential of pulmonary vascular endothelial cells. (3) Reduces the levels of pro-inflammatory cytokines (IL-6 and TNF-α) in lung tissues, effectively suppressing the inflammatory response in a hyperoxic environment. (4) Reduced the proportion of inflammation-associated lymphocytes (NK cells, B cells, CD8^+^ T cells and CD4^+^ T cells). (5) Promoted the recovery of alveolar epithelial cells (AT1/AT2 cells).

Excessive oxygen concentration and partial pressure of breathed oxygen, or prolonged exposure, can result in oxygen toxicity, which may be life-threatening in severe instances (Alva et al., 2023). Despite the extensive application of hyperbaric oxygen in medicine for many ailments, vigilance regarding the potential for medical errors remains essential. Certain investigation by Braun et al. (2018) have demonstrated that intraperitoneal administration of exosomes produced from mesenchymal stromal cells can safeguard the lung from hyperoxia-induced bronchopulmonary dysplasia, with the mechanism manifesting in two facets. As shown in Figure 2, (refer to Table 1) exosomes exert a notable anti-inflammatory effect by suppressing the M1/M2 polarization of macrophages and down-regulating the inflammatory cascade. Conversely, exosomes can stimulate the creation of tubular networks in pulmonary vascular endothelial cells by delivering and targeting vascular endothelial growth factor, thus facilitating neovascularization and enhancing tissue healing and homeostasis restoration. Likewise, as shown in Figure 2, additional study by Yin et al. (2024) indicated that MSC-Exos could diminish the percentage of inflammation-associated lymphocytes (NK cells, B cells, CD8^+^T cells, and CD4^+^T cells) and the concentrations of pro-inflammatory cytokines (IL-6 and TNF-α) in lung tissue, thereby effectively suppressing the inflammatory response in a hyperoxic environment. It also facilitates the proliferation of alveolar epithelial cells (AT1/AT2 cells), underscoring its pivotal function in lung tissue regeneration. Together, these mechanisms demonstrate the numerous protective benefits of exosomes in lung function repair through the dual effects of tissue regeneration and immune regulation, and they offer a strong scientific foundation for the potential benefits of exosomes in combination with hyperbaric oxygen.

### 4.4 Synergistic strengthening effect of hyperbaric oxygen combined with exosomes

#### 4.4.1 Synergistic inhibition of inflammatory response

Following trauma, the infiltration of macrophages and other inflammatory cells into the wounded area, together with their release of proinflammatory cytokines such as TNF-α and IL-1β, can worsen tissue damage and impede injury recovery (Biglari et al., 2015; Boato et al., 2013). Hyperbaric oxygen has a synergistic effect with exosomes’ immunomodulatory action, inhibiting both the infiltration of inflammatory cells and the production of inflammatory factors (Alenazi et al., 2021; Shi et al., 2025). Numerous studies have also demonstrated that the combined anti-inflammatory effects of the two medications are more potent than those of either medication alone. The study by Hjazi et al. (2024) demonstrated that the level of TNF-α following treatment with exosomes derived from human menstrual blood stem cells and hyperbaric oxygen was significantly lower than that of exosomes (*p* < 0.01) or hyperbaric oxygen (*p* < 0.05) alone, and the level of IL-1β was significantly lower than that of exosomes alone (*p* < 0.05). The work by Amiri et al. (2025) also demonstrated that in a rat model of sciatic nerve damage, exosomes derived from human placental mesenchymal stem cells (hpMSCs-Exos) combined with hyperbaric oxygen treatment significantly reduced the levels of TNF-α (*p* < 0.001 and *p* < 0.01), IL-1β (*p* < 0.01 and *p* < 0.05), and interferon-γ (*p* < 0.01 and *p* < 0.05). Compared to exosomes or hyperbaric oxygen alone, the effect is superior.

Together, the two have a synergistic impact that increases the expression of anti-inflammatory factors while also suppressing pro-inflammatory factors. Interferon-γ, TNF-α, and IL-1β expression levels can be markedly downregulated by the anti-inflammatory cytokine IL-10, which has been demonstrated in studies to be one of the most important elements in promoting neuronal survival following damage (Patilas et al., 2024). Cheshmi et al. (2023) and Jafari et al. (2023) demonstrated that hpMSCs-Exos in conjunction with hyperbaric oxygen resulted in a more pronounced elevation of IL-10 gene expression following traumatic spinal cord injury and spinal cord ischemia-reperfusion injury compared to the intervention approach alone (all *p* < 0.05).

#### 4.4.2 Synergistic reduction of oxidative stress

Early post-traumatic oxidative stress is a significant pathogenic occurrence in the affected region (Yu et al., 2023). The body’s antioxidant system, including glutathione, catalase, and superoxide dismutase, plays a crucial role in mitigating oxidative stress. Excessive production of reactive oxygen species following injury will disrupt the antioxidant system, activate apoptosis-related proteins, and trigger cellular death (Valko et al., 2007). Consequently, regulating oxidative stress is crucial in mitigating the extent of damage. Several research have indicated that hyperbaric oxygen, in conjunction with exosomes, can elevate the biological levels of glutathione, catalase, and superoxide dismutase, hence demonstrating a synergistic effect in augmenting antioxidant capability (Amiri et al., 2025; Cheshmi et al., 2023; Hjazi et al., 2024; Jafari et al., 2023). Conversely, it can diminish the amounts of reactive oxygen species, propylene glycol, and other oxidative agents, demonstrating a synergistic suppression of the oxidative stress response (Amiri et al., 2025; Cheshmi et al., 2023; Hjazi et al., 2024; Jafari et al., 2023). The aforementioned research have established that combination interventions are superior to any singular approach.

#### 4.4.3 Synergetically protect the nervous tissue

Numerous studies indicate that exosomes in conjunction with hyperbaric oxygen therapy can markedly enhance neuronal survival and proliferation. Jafari et al. (2023) indicated that the numerical density of neurones dramatically increased following the administration of hpMSCs-Exos in conjunction with hyperbaric oxygen therapy in a rat model of spinal cord ischemia-reperfusion injury (*p* < 0.001). Cheshmi et al. (2023) shown that, in the treatment of severe spinal cord injury, the combination of hpMSCs-Exos with hyperbaric oxygen therapy yielded a significantly higher neuronal count compared to either treatment alone (*p* < 0.05). The stereological evaluation of the spinal cord at the lesion site, employing Cavalier’s method, demonstrated that the average total spinal cord volume in the combined treatment group was approximately double that of the control group, and the total spinal cord volume in the combined treatment group was significantly greater than that of the single treatment group (all *p* < 0.05). Amiri et al. (2025) also found that, when hyperbaric oxygen combined with hpMSCs-Exos synergistically promoted the recovery of sciatic nerve injury, compared with hyperbaric oxygen or exosomes intervention alone, the sciatic nerve volume after combined treatment was larger (both, *p* < 0.05), thicker myelin sheath (*p* < 0.01 and *p* < 0.05), and more nerve fibers (*p* < 0.05 and *p* < 0.01). Furthermore, the study utilising Cavalier’s approach revealed that the overall spinal anterior horn volume was greater with combination therapy compared to exosomes (*p* < 0.05) or hyperbaric oxygen (statistically insignificant) administered alone. All these studies demonstrated that the mechanism underlying enhanced neuronal proliferation and survival was associated with the reduction of the apoptosis-related protein caspase-3 activity through the combination administration of the two medicines.

## 5 Application of hyperbaric oxygen combined with exosomes in promoting tissue repair

### 5.1 Diabetic wound

Chronic hyperglycemia in diabetic individuals can cause peripheral nerve and microvascular damage, leading to diminished angiogenesis, heightened oxidative stress, infections, and other risk factors, ultimately resulting in the development of diabetic refractory wounds (Lin et al., 2023). Hyperbaric oxygen is an efficacious treatment for diabetic wounds (OuYang et al., 2024), primarily functioning by enhancing the hypoxic environment, stimulating cell proliferation and angiogenesis, and diminishing oxidative stress and inflammatory responses (Capó et al., 2023). Recent evidence has proved that exosomes are one of the key secreted products that mediate intercellular communication and accelerate wound healing (Hade et al., 2022). A study by Yin et al. (2024) demonstrated that exosomes released from human umbilical vein endothelial cells subjected to hyperbaric oxygen were co-cultured with white adipose tissue in culture medium, resulting in the Browning of white adipose tissue, which transformed into beige adipocytes after phagocytosis of the endothelial cell-derived exosomes. Beige adipocytes can induce M2 polarization in macrophages via elevated release of neuregulin 4, suppress inflammatory responses, and enhance fibroblast proliferation and angiogenesis, thereby facilitating tissue regeneration and enhancing diabetic wound healing (Cai et al., 2024). The *in vitro* findings of this study indicated that hyperbaric oxygen-induced exosomes from vascular endothelial cells may enhance diabetic wound healing by promoting the browning of adipocytes.

Moreover, exosomes produced from stem cells might expedite the healing of diabetic wounds by transporting therapeutic growth factors and microRNAs (Qiao et al., 2023), however, the hypoxic conditions following injury may impair the transportation effectiveness of exosomes within cells (Tong et al., 2023). Consequently, the concurrent delivery of exosomes and oxygen is notably significant. Numerous research have addressed the constraint of hypoxia by developing a novel method including exosomes-coated oxygen nanoparticles, which have demonstrated significant therapeutic efficacy in improving diabetic wound healing (Han et al., 2024; Zhang, et al., 2023a). This therapy technique necessitates advanced technologies and substantial costs, hindering its widespread clinical implementation. Hyperbaric oxygen, as an economical and straightforward oxygen supply method, has been extensively utilized in clinical settings. Its integration with exosomes presents significant potential for the treatment of diabetic wounds, representing a novel avenue for future research.

### 5.2 Nervous system diseases

#### 5.2.1 Traumatic spinal cord injury

Traumatic spinal cord injury denotes the impairment of spinal cord architecture and neural function due to external forces, leading to dysfunction in sensory, motor, and autonomic nerves (Grijalva-Otero and Doncel-Pérez, 2024). Following an injury, a series of molecular and cellular processes ensues, including apoptosis, gliosis, oxidative stress, and inflammation (Hu et al., 2023). Secondary injury can aggravate the disease, potentially resulting in the loss of sensory and motor functions at or below the site of spinal cord injury. To avert the propagation of secondary injury, prompt therapy following an injury to facilitate the healing of compromised tissues is the most efficacious strategy for traumatic spinal cord injury (Adegeest et al., 2023). Research has shown that early hyperbaric oxygen treatment following spinal cord injury can markedly diminish the expression of pro-inflammatory proteins (such as TNF-α and IL-1β) and enhance the production of anti-oxidative stress markers (such as superoxide dismutase), while also decreasing neuronal death and gliosis (Sunshine et al., 2023). Stem cell treatment is recognized for its advantageous impact on spinal cord injuries. Yin et al. (2022) investigated a rat model of acute traumatic spinal cord injury using hyperbaric oxygen in conjunction with adipose-derived mesenchymal stem cells, revealing that the combined therapy exhibited a more pronounced effect than individual treatments. This treatment approach markedly diminishes oxidative stress and inflammatory signals by collaboratively down-regulating inflammatory responses and cellular stress pathways, including PI3K/AKT/mTOR, thus safeguarding spinal cord components. Recent research indicates that the advantageous effects of stem cells on spinal cord injury may be facilitated by the paracrine release of exosomes, rather than their capacity to specialize into various cell types (Khalatbary, 2021). Various microRNAs included in stem cell-derived exosomes can significantly diminish neuronal apoptosis, enhance angiogenesis, suppress inflammatory responses, and facilitate axon regeneration by modulating many signaling pathways, consequently augmenting functional recovery following spinal cord injury (Hwang et al., 2023).

Consequently, Cheshmi et al. (2023) conducted the first study on the combined application of hyperbaric oxygen and exosomes in a rat model of traumatic spinal cord injury. Ninety male rats were randomly divided into 5 groups. The rats in the combined treatment group (n = 18) were injected with 200 μ hpMSCs-Exos through the tail vein 30 min after the establishment of traumatic spinal cord injury model and treated with Hyperbaric oxygen (2.5ATA, 90 min, a total of 3 times). The study found that compared with hyperbaric oxygen or exosomes therapy alone, the Basso-Beattie-Bresnehan test scores of rats in the combined treatment group were higher on day 1 (*p* < 0.05 and *p* < 0.05), day 3 (*p* < 0.05 and *p* < 0.05), day 7 (*p* < 0.05 and *p* < 0.05) and day 14 (*p* < 0.05 and *p* < 0.05) after injury. The narrow beam walking test had higher scores on day 1 (*p* < 0.05 and *p* < 0.05), day 7 (statistically insignificant and *p* < 0.05), and day 14 (*p* < 0.05 and *p* < 0.05) after injury. In addition, the results of electromyography latency test showed that the latency was significantly shorter after combined treatment (*p* < 0.05 and *p* < 0.05).

hpMSCs-Exos exhibit minimal immunogenicity and have been utilized in preliminary clinical trials concerning tissue regeneration (Zamanian et al., 2024). The expansion of human placental mesenchymal stem cells is constrained, with a doubling time approximately 40 h. Prolonged culturing may induce persistent alterations in mesenchymal stem cells (Kim and Park, 2017). To address the proliferative problem, Meng et al. (2007) first found human menstrual blood stem cells in 2007. The cells offer benefits such as non-invasive isolation, periodic acquisition, minimal immunological rejection, and the absence of ethical concerns. The characteristic of a rapid proliferation rate, with a doubling period of approximately 19.4 h and a doubling rate double that of human placental mesenchymal stem cells, renders them a prospective therapeutic alternative (Khoury et al., 2014). In this context, Hjazi et al. (2024) examined the effectiveness of human menstrual blood stem cell-derived exosomes in conjunction with hyperbaric oxygen (2.5 ATA, 90 min, 3 sessions) in a rat model of traumatic spinal cord injury, adhering to a methodology aligned with the aforementioned study. The study’s results indicated that the Basso-Beattie-Bresnehan test scores for the combination treatment were considerably superior to those for exosomes treatment alone on day 1 (*p* < 0.01), day 7 (*p* < 0.05), and day 14 (*p* < 0.05) post-injury. In comparison to hyperbaric oxygen therapy alone, the combination treatment demonstrated improvement; nevertheless, the difference was not statistically significant. The combined therapy exhibited a trend towards reduced EMG latency compared to each intervention individually; however, the difference did not achieve statistical significance. Human menstrual blood stem cells do not express MHC-II cell surface proteins that are recognized by CD4^+^T cells, hence exhibiting minimal immunogenicity and reducing the likelihood of immunological rejection (Khoury et al., 2014). Following injection into rats, exosomes generated from stem cells were able to enhance the expression of MHC-II in response to interferon-γ activation, hence synergistically activating T cells alongside costimulatory molecules (CD80/CD86) (Butticè et al., 2006). Furthermore, hyperbaric oxygen has been shown to enhance MHC-II biosynthesis in exosomes (Yin et al., 2024). Therefore, the above reasons may have resulted in a reduced delivery efficiency of human menstrual blood stem cell-derived exosomes in the rat model, resulting in a statistically insignificant difference after combination treatment. The limited sample size, comprising only 6 out of 18 rats per group for behavioral function testing, constrained the efficacy of Tukey’s one-way analysis of variance (ANOVA) with a *post hoc* test, potentially resulting in biologic differences not achieving the significance threshold of *p* < 0.05 due to substantial standard deviations. Consequently, future preclinical trials must be conducted to increase the sample number and assess the efficacy disparities of exosomes derived from various sources in conjunction with hyperbaric oxygen therapy.

#### 5.2.2 Spinal cord ischemia-reperfusion injury

Spinal cord ischemia-reperfusion injury is a non-traumatic spinal cord injury frequently encountered following abdominal aortic surgery. Severe diseases may result in paraplegia; however, existing therapeutic modalities have not produced consistent clinical outcomes (Xie et al., 2024). Jafari et al. (2023) employed a combined therapy strategy to investigate a rat model of spinal cord ischemia-reperfusion injury, building on the efficacy of hyperbaric oxygen in conjunction with exosomes in a model of traumatic spinal cord injury. This study involved the random allocation of 80 rats into five groups. In the combined treatment group (n = 16), spinal cord ischemia-reperfusion injury was caused by aortic occlusion for 60 min, succeeded by hyperbaric oxygen therapy (2.5 ATA, 60 min each, administered twice) during both ischemia and reperfusion phases. hpMSCs-Exos (20 μg) were administered via the tail vein 30 min post-model establishment. The combined treatment markedly reduced oxidative stress and inflammation, and diminished apoptosis during spinal cord ischemia-reperfusion injury. It can significantly enhance neurological performance and collaboratively diminish histopathological damage by safeguarding neuronal viability and suppressing spinal gliosis. The motor deficit index test indicated that the combination treatment resulted in a substantial decrease in the motor deficit index score at 6 h (*p* < 0.01 and *p* < 0.05), 12 h (*p* < 0.05 and *p* < 0.05), and 48 h (*p* < 0.05 and *p* < 0.05) post-operation, in comparison to the individual interventions of exosomes and hyperbaric oxygen. The construction of this rat model fully simulates the unexpected events during abdominal aortic surgery, which is in line with the logic of clinical perioperative intervention. Evaluating its safety is the key direction of future research.

#### 5.2.3 Sciatic nerve injury

Sciatic nerve injury is a form of peripheral nerve injury characterized by structural or functional damage to the sciatic nerve due to trauma, compression, inflammation, or metabolic disorders, leading to sensory, motor, and autonomic dysfunction in the lower extremities (Wang et al., 2024). Numerous studies indicate that hyperbaric oxygen enhances peripheral nerve damage recovery by promoting endogenous neurogenesis via mechanisms including anti-inflammation and oxidative stress reduction (Brenna et al., 2023). Exosomes have demonstrated significant potential in the therapy of peripheral nerve damage (Namini et al., 2023). Considering the synergistic augmentation of hyperbaric oxygen and exosomes in ameliorating central nerve injury (spinal cord injury), Amiri et al. (2025) further investigated the effectiveness of their combination on peripheral nerve injury. Seventy-five rats were randomly allocated into five groups during the trial. In the combined treatment group (n = 15), 200 μg of hpMSCs-Exos was administered into the gastrocnemius muscle of the rats following the completion of sciatic nerve injury, in conjunction with hyperbaric oxygen therapy (2 ATA, 60 min, 7 sessions). Post-intervention, the behavioral assessment indicated that the delay of electromyography was considerably reduced following combination therapy compared to exosomes treatment alone (*p* < 0.05). In comparison to the administration of exosomes and hyperbaric oxygen independently, the sciatic nerve function index score was elevated following the combination treatment on days 7 (*p* < 0.001 and *p* < 0.01), 14 (*p* < 0.01 and *p* < 0.05), 21 (*p* < 0.01 and *p* < 0.05), and 28 (*p* < 0.01 and *p* < 0.05).

The amalgamation of hpMSCs-Exos and hyperbaric oxygen therapy exhibits a substantial therapeutic impact in mitigating inflammation, oxidative stress, and tissue alterations following nerve injury, hence establishing a critical scientific foundation for the clinical use of this approach. Nonetheless, it is crucial to recognize the therapeutic constraints of hyperbaric oxygen therapy. Hyperbaric oxygen therapy may be impractical for patients in intensive care units due to the requirement for specialist equipment and meticulous monitoring. These practical constraints underscore the challenges of implementing this therapy in a clinical environment.

### 5.3 Vascular diseases

**FIGURE 3:**
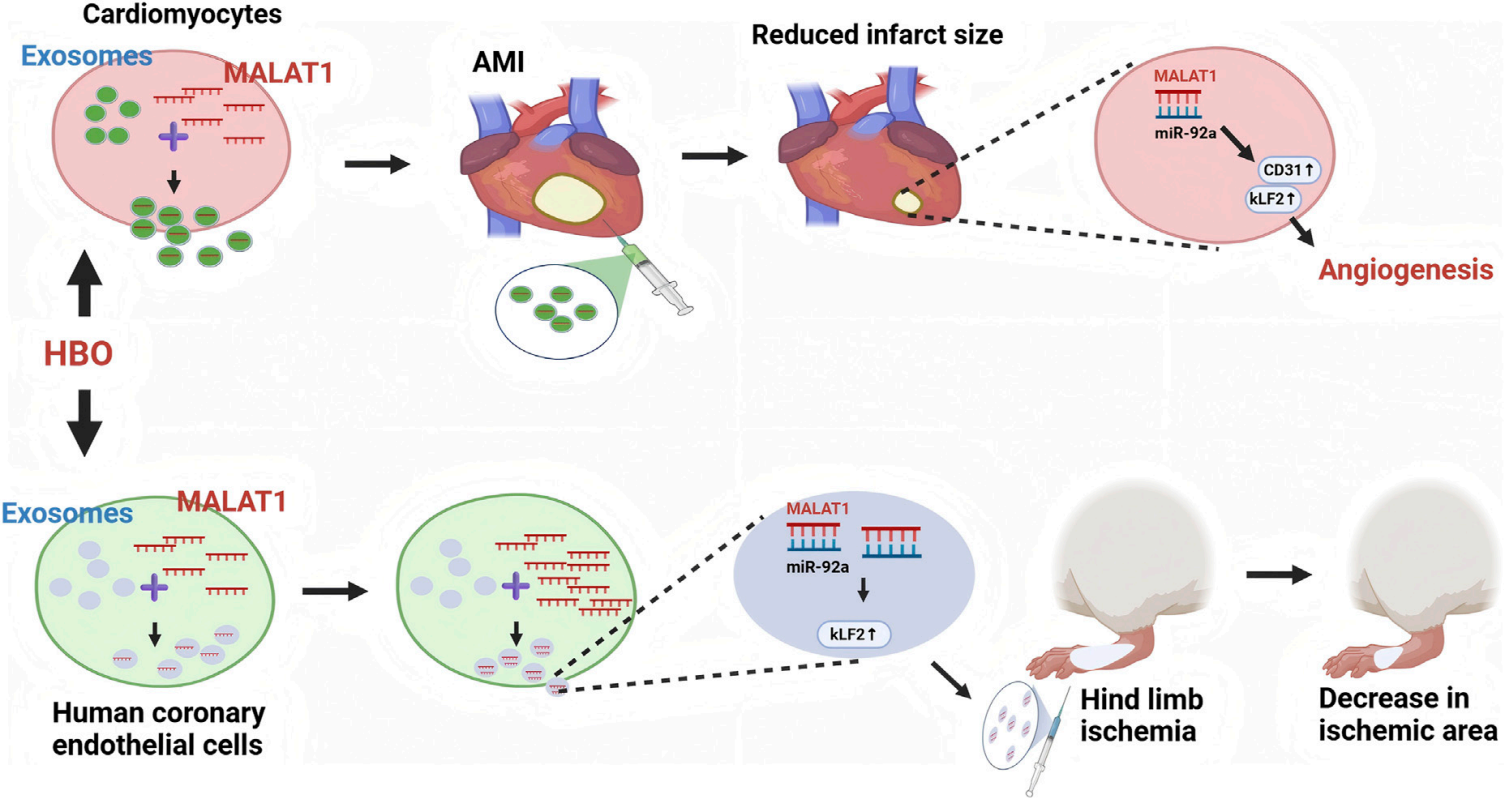
Hyperbaric oxygen stimulates HCAECs-derived exosomes to suppress miR-92a expression by upregulating MALAT1, thereby counteracting the inhibitory effect of miR-92a on KLF2 expression and promoting angiogenesis. Hyperbaric oxygen combined with exosomes facilitated the interaction between MALAT1 and miR-92a in rat cardiomyocytes, enhancing the release of KLF2 and the endothelial cell adhesion molecule CD31, which resulted in a substantial decrease in myocardial infarct size.

Vascular disease is a significant contributor to morbidity and death globally; thus, identifying effective non-invasive treatment methods is crucial to mitigate adverse outcomes (Balta, 2021). Shyu et al. (2019) initially discovered that hyperbaric oxygen induces exosomes containing MALAT1, which can enhance angiogenesis. In this investigation, HCAECs were subjected to a minor hyperbaric oxygen chamber (2.5 ATA, 60 min, twice) for 2 days, after which exosomes were extracted from the cell culture medium utilizing a whole exosomes isolation reagent. Subsequently, 70 μg of exosomes were transfected into a rat model of right femoral artery ligation wound via a low-pressure accelerated gene gun (n = 6). Post-treatment, laser Doppler perfusion imaging revealed that exosomes induced by hyperbaric oxygen markedly enhanced blood flow in the ischemic hind limb of rats, with the blood flow ratio (ischemic hind limb/normal hind limb) significantly exceeding that of the control group (*p* < 0.001). Additionally, a unique mechanism was hypothesized, as shown in Figure 3, whereby hyperbaric oxygen stimulates HCAEC-derived exosomes to suppress miR-92a expression by upregulating MALAT1, thereby counteracting the inhibitory effect of miR-92a on KLF2 expression and promoting angiogenesis.

This study’s findings indicate that another investigation by the same research team identified the therapeutic potential of hyperbaric oxygen-induced exosomes containing MALAT1 for myocardial infarction and further elucidated the mechanism of their combined application to enhance angiogenesis (Shyu et al., 2020). In this study, 70 μg of isolated cardiomyocyte-derived exosomes were administered into the peri-infarct region of a rat myocardial infarction model (n = 5) by a low-pressure accelerated gene gun in three separate instances, in conjunction with hyperbaric oxygen therapy (2.5 ATA, 60 min, a total of 14 sessions). As shown in Figure 3, the co-administration of hyperbaric oxygen and exosomes facilitated the interaction between MALAT1 and miR-92a in rat cardiomyocytes, enhancing the release of KLF2 and the endothelial cell adhesion molecule CD31, which resulted in a substantial decrease in myocardial infarct size (p < 0.01). Moreover, the cardiac ejection fraction was increased by 14% on average compared with non-hyperbaric oxygen-induced exosomes. The function of MALAT1 in myocardial infarction, as shown by prior research, is contentious. Certain investigation by Guo et al. (2019) have indicated the advantageous role of MALAT1 following myocardial infarction; nevertheless, another study by Gong et al. (2019) reported that elevated MALAT1 levels induced myocardial cell death in mice with myocardial infarction, and the rationale for this discrepancy remains inadequately elucidated. These findings offer substantial evidence for clarifying the beneficial effects of MALAT1 during myocardial infarction, and the integration of MALAT1-enriched exosomes with hyperbaric oxygen therapy may represent a promising therapeutic approach for enhancing angiogenesis.

## 6 Safety and transformation barrier analysis of hyperbaric oxygen combined with exosomes in promoting tissue repair

### 6.1 Safety analysis of hyperbaric oxygen combined with exosomes to enhance tissue regeneration

The evaluation of the safety of hyperbaric oxygen in conjunction with exosomes to enhance tissue repair is fundamental to clinical application. This combined therapy technique demonstrates evident synergistic effects in anti-inflammatory, anti-oxidative, pro-angiogenic, and neuroprotective domains, alongside minimal toxicity in animal trials; however, potential human hazards must not be overlooked. A systematic review and meta-analysis of the adverse effects of hyperbaric oxygen therapy included 24 randomized controlled trials with 1,497 subjects, revealing an overall adverse effect rate of 30.11%, with ear discomfort being the most prevalent side effect (113 cases). Nonetheless, when the hyperbaric oxygen treatment regimen (≤10 sessions) or chamber pressure (<2.5 ATA) was adequately regulated, the occurrence of adverse effects was markedly diminished (Zhang, et al., 2023b). Therefore, the hyperbaric oxygen dose is particularly important. As nanoscale vesicles, the immunogenicity of exosomes is the key factor. Exosomes derived from autologous cells are generally well tolerated, but allogeneic or xenogeneic sources may trigger immune rejection or allergic reactions. A review of exosomes immunogenicity points to the potential risk that exosomes surface proteins, such as MHC complexes, may activate T cell responses, leading to autoimmune diseases (Théry et al., 2018). In addition, contamination during exosomes extraction and purification, such as viral or bacterial endotoxins, may also introduce infection risk (Théry et al., 2018). Furthermore, in a hyperbaric oxygen environment, the stability of exosomal membranes may be compromised, resulting in the release of contents and the activation of unforeseen cellular signaling pathways, hence exacerbating toxicity. Consequently, while the approach of utilizing hyperbaric oxygen in conjunction with exosomes to enhance tissue healing is interesting, its safety requires additional investigation.

### 6.2 Analysis of transformation barriers in tissue repair promoted by hyperbaric oxygen combined with exosomes

The effective delivery of exosomes is the major bottleneck in clinical translation. According to pharmacokinetic data, exogenous exosomes have a short residence time in the human circulation and are susceptible to clearance by macrophages associated with organs of the mononuclear phagocytic system (liver, spleen, and lung) (Parada et al., 2021). In the hyperoxic environment created by hyperbaric oxygen, the lipid membrane of exosomes may undergo oxidation and degradation, hence diminishing their bioavailability (Lis et al., 2011). Furthermore, existing delivery methods (including intravenous or topical administration) exhibit inefficiency, with about 10%–20% of exosomes attaining the target regions (Lener et al., 2015). The exosomes delivery protocol for the aforementioned investigation in the myocardial infarction model involves injection into the infarct border zone, which poses challenges for practical translation. To improve stability in tissue repair, it is essential to produce tailored exosomes, including surface-modified targeting ligands, or biomaterial carriers, such as hydrogels (Tran et al., 2020). The pressure gradient of hyperbaric oxygen may disrupt these delivery routes, resulting in non-specific distribution and possible toxicity. Consequently, imaging techniques like fluorescent tagging should be a crucial instrument for evaluating delivery efficacy in clinical trials.

The effective dose of exosomes in animal research (about 10^9^–10^10^ exosomes per kilogram of body weight) presents challenges for direct scaling to humans (Gupta et al., 2021). Variations in human body size and metabolism may necessitate a 10- to 100-fold augmentation in dosage; nevertheless, excessive dosing poses a danger of immunotoxicity or thrombosis, while insufficient dosing may prove ineffectual. The interplay between hyperbaric oxygen therapy and variations in oxygen partial pressure can influence the kinetics of exosomes release, hence complicating dose optimization. Consequently, clinical translation necessitates pharmacokinetic and pharmacodynamic modeling to replicate human dosages, accompanied by dose-escalation trials to mitigate risk. In addition, there are physiological differences between animal models and humans, including the immune system, oxygen metabolism, and tissue repair mechanisms (Dietz and Schwab, 2017; Nardone et al., 2017). For example, the high metabolic rate in mice may exaggerate the oxygenation benefits of hyperbaric oxygen (Mortola, 2023). Furthermore, the majority of prior studies administered hyperbaric oxygen or exosomes interventions shortly after model building to achieve optimal outcomes. In clinical practice, treatment is typically commenced during the subacute or chronic phase of injury, and this timely approach is constrained by certain objective characteristics that are challenging to mimic in a clinical environment. These discrepancies may result in diminished efficacy or unforeseen adverse outcomes in clinical trials. Consequently, it is imperative to assess the effectiveness of delayed intervention in large animal models (such as non-human primates) prior to translation, optimize the timing of intervention to confirm its efficacy, and incorporate omics data (such as transcriptome analysis) to bridge the interspecies gap.

In conclusion, while hyperbaric oxygen combined with exosomes demonstrates enhanced efficacy in facilitating tissue repair in animal models, the application of this combination in human clinical trials encounters several constraints, including exosomes delivery methods, dosage scaling, interspecies variations, and timing of intervention. These problems must be resolved by meticulous preclinical optimization and phase I/II trials to guarantee a balance between efficacy and safety.

## 7 Discussion and outlook

The investigation of hyperbaric oxygen combined with exosomes for enhancing tissue repair possesses a robust scientific foundation and significant therapeutic applicability. Currently, an increasing number of research indicate that hyperbaric oxygen therapy and exosomes exhibit beneficial synergistic effects in the repair of various forms of tissue damage (refer to Supplementary Material). Hyperbaric oxygen markedly enhances tissue repair by augmenting local oxygen availability, facilitating angiogenesis, and diminishing oxidative stress. Conversely, exosomes facilitate tissue regeneration by modulating immunological responses, enhancing cell proliferation and migration, and expediting wound healing. The mechanisms of action strongly endorse the viability of hyperbaric oxygen in conjunction with exosomes for clinical treatment. Moreover, current research has validated that hyperbaric oxygen therapy, in conjunction with exosomes, exhibits a synergistic enhancement in anti-inflammatory, antioxidant, and neuroprotective effects, demonstrating remarkable efficacy across various disease models, including diabetic wounds, neurological disorders, and vascular diseases. Nonetheless, critical issues remain about safety and translational hurdles, including the prevention of adverse effects from hyperbaric oxygen therapy, the efficacy of exosomes delivery, and interspecies variations. Furthermore, the pathophysiological mechanisms and oxygen requirements of various diseases varies; thus, the specific method for hyperbaric oxygen therapy combined with exosomes treatment should be tailored to the disease’s peculiarities. Future research should concentrate on examining the impacts of various treatment protocols on tissue regeneration and identifying the most effective treatment combinations and dosages. From a clinical translation standpoint, while hyperbaric oxygen therapy is extensively utilized for traumatic conditions, clinical investigations into the combination of hyperbaric oxygen and exosomes have yet to be conducted. A substantial number of clinical trials are required to guarantee its safety and efficacy in clinical application. Moreover, the production costs, quality assurance, and standardized extraction and storage techniques of exosomes are practical challenges that require resolution. Consequently, the establishment of pertinent standardized procedures and norms to guarantee the stability and uniformity of exosomes across various application contexts would be essential for their clinical advancement.

Anticipating the future, the ongoing advancements in bioengineering and nanotechnology, the enhancement of exosomes delivery systems, the innovation of integrated treatment modalities, and the identification of pertinent biomarkers will significantly bolster the clinical application of hyperbaric oxygen therapy in conjunction with exosomes therapy. Treatment strategies founded on personalized medicine will customize optimal treatment plans for patients with various diseases, thereby enhancing therapeutic efficacy and minimizing potential negative effects. Nevertheless, the current evidence predominantly relies on animal models and *in vitro* studies, with a deficiency of extensive human clinical data. Future research should include randomized controlled trials to assess the long-term efficacy and safety of combination therapy, including tissue healing rates, inflammatory marker levels, and adverse event occurrences. Simultaneously, incorporating multi-omics methodologies, including transcriptomics and proteomics, to clarify the molecular interactions between hyperbaric oxygen and exosomes may aid in uncovering potential synergistic pathways and inform the creation of innovative intervention techniques. Furthermore, given the heterogeneity of exosomes—where exosomes from various cell types exhibit varying repair capabilities—subsequent research should investigate the ideal ratio of distinct exosomes sources to hyperbaric oxygen. This enhances treatment specificity and may possibly diminish the likelihood of allogeneic rejection. Advancing the establishment of international consensus guidelines will guarantee that clinical trials of combination medicines adhere to Good Clinical Practice (GCP) standards and facilitate their uniform implementation globally.

The investigation of hyperbaric oxygen in conjunction with exosomes holds significant potential, particularly in tissue repair, offering novel concepts and treatment approaches. Subsequent study should persist in investigating its mechanisms, refining treatment methodologies, and further validating its efficacy and safety in clinical settings, thereby paving a new avenue for the treatment of associated disorders. This integrated approach, through multidisciplinary collaboration, is anticipated to be a significant advancement in regenerative medicine, expediting the transition from fundamental research to clinical application.
