# Correction: Hyperbaric oxygen in combination with exosomes: a new strategy to promote tissue repair

**DOI:** 10.3389/fbioe.2025.1727054

**Published:** 2025-11-07

**Authors:** Shengchao Zhang, Jiaqi Zhou, Jia Liu, Tong Li, Yong Liu, Yuling Gao

**Affiliations:** Department of Rehabilitation Medicine, The First Affiliated Hospital of Dalian Medical University, College of Health-Preservation and Wellness, Dalian Medical University, Dalian, China

**Keywords:** hyperbaric oxygen, exosomes, small extracellular vesicles, tissue repair, regenerative medicine

In the published article, there was a mistake in the affiliations. The author affiliation is shown as “^1^Department of rehabilitation medicine, the First Affiliated Hospital of Dalian Medical University, Dalian, China, ^2^ College of Health-Preservation and Wellness, Dalian Medical University, Dalian, China”.

The correct version should be “^1^Department of Rehabilitation Medicine, the First Affiliated Hospital of Dalian Medical University, College of Health-Preservation and Wellness, Dalian Medical University, Dalian, China”.

The original article has been updated.

